# Supplementary material for: Comparison of Ovarian Morphology and Follicular Disturbances between Two Inbred Strains of Cotton Rats (Sigmodon hispidus)
Source: Animals (Basel). 2021 Jun 12;11(6):1768. doi: 10.3390/ani11061768 (PMC8231567; doi:10.3390/ani11061768)
Supplement: Supplementary file 1 [file animals-11-01768-s001.zip › animals-1251892-supplementary.pdf]

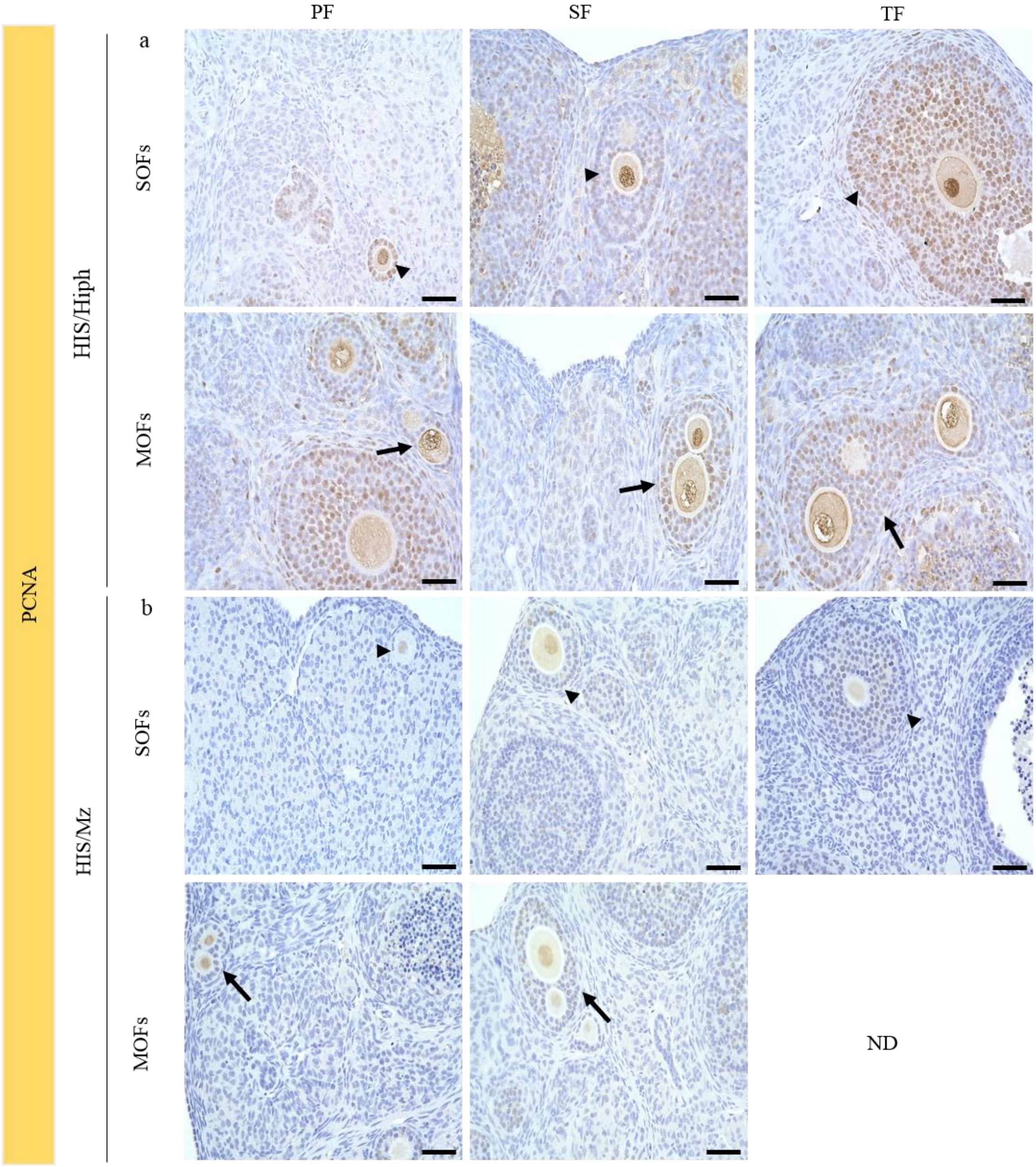


**Figure S1: Evaluation of proliferative activity of oocytes in HIS/Hiph and HIS/Mz**

**(a and b)** Immunohistochemistry for PCNA. PCNA-positive oocytes in single oocyte follicles (SOFs) (arrowhead) and multi-oocyte follicles (MOFs) (arrows) were observed in the primary follicles (PFs), secondary follicles (SFs), and tertiary follicles (TFs) in HIS/Hiph (a). PCNA-positive SOFs and MOFs (weak expression) were observed in PFs, SFs, and TFs in HIS/Mz (b). Scale bars 100 μm. ND: Not detected.
